# Left Right Judgement Task and Sensory, Motor, and Cognitive Assessment in Participants with Wrist/Hand Pain

**DOI:** 10.1155/2018/1530245

**Published:** 2018-08-26

**Authors:** René Pelletier, Daniel Bourbonnais, Johanne Higgins, Maxime Mireault, Michel Alain Danino, Patrick G. Harris

**Affiliations:** ^1^Sciences de la Réadaptation, École de Réadaptation, Faculté de Médecine, Université de Montréal, Montréal (Québec), Canada H3C 3J7; ^2^École de Réadaptation, Faculté de Médecine, Université de Montréal, C.P. 6128, Succursale Centre-Ville, Montréal (Québec), Canada H3C 3J7; ^3^Researcher, Centre for Interdisciplinary Research in Rehabilitation of Greater Montreal (CRIR), Canada; ^4^Professeur Agrégé Université de Montréal, Chef du Service de Chirurgie Plastique du Centre Hospitalier Université de Montréal (CHUM), 850 rue St-Denis Pav. S-Local S02-128 Montréal (Québec), Canada H2X 0A9; ^5^Service de Chirurgie Plastique, Département de Chirurgie du Centre Hospitalier de l'Université de Montréal (CHUM), 1000 rue Saint-Denis (Québec), Canada H2X 0C1

## Abstract

The Left Right Judgement Task (LRJT) involves determining if an image of the body part is of the left or right side. The LRJT has been utilized as part of rehabilitation treatment programs for persons with pain associated with musculoskeletal injuries and conditions. Although studies often attribute changes and improvement in LRJT performance to an altered body schema, imaging studies suggest that the LRJT implicates other cortical regions. We hypothesized that cognitive factors would be related to LRJT performance of hands and feet and that sensory, motor, and pain related factors would be related to LRJT in the affected hand of participants with wrist/hand pain. In an observational cross-sectional study, sixty-one participants with wrist/hand pain participated in a study assessing motor imagery ability, cognitive (Stroop test), sensory (Two-Point Orientation Discrimination, pressure pain thresholds), motor (grip strength, Purdue Pegboard Test), and pain related measures (West Haven Yale Multidimensional Pain Inventory) as well as disability (Disability of the Arm, Shoulder and Hand). Multiple linear regression found Stroop test time and motor imagery ability to be related to LRJT performance. Tactile acuity, motor performance, participation in general activities, and the taking of pain medications were predictors of LRJT accuracy in the affected hand. Participants who took pain medications performed poorly in both LRJT accuracy (p=0.001) and reaction time of the affected hand (p=0.009). These participants had poorer cognitive (p=0.013) and motor function (p=0.002), and higher pain severity scores (p=0.010). The results suggest that the LRJT is a complex mental task that involves cognitive, sensory, motor, and behavioural processes. Differences between persons with and without pain and improvement in LRJT performance may be attributed to any of these factors and should be considered in rehabilitation research and practice utilizing this task.

## 1. Introduction

The Left Right Judgement Task (LRJT) involves determining, as accurately and as quickly as possible, if an image of a body part is of the left or right side. LRJT performance differences between persons with and without pain have been hypothesized to reflect changes in central nervous system processing, errors in judgement, and changes in bodily representations [1]. Studies have demonstrated that the LRJT, as part of a treatment program in persons with pain, may result in statistically and clinically significant improvement in pain and function [2–5].

Studies involving the LRJT have been performed with persons experiencing pain associated with musculoskeletal injuries and conditions with variable findings. These include no changes in LRJT performance [6], localised [7, 8], bilateral [9, 10], and hemilateral changes [11, 12]. Differences in LRJT performance are often attributed to differences in the body schema [12–17] and attentional bias [18, 19]. The body schema refers to the internal representation of the body in peri-personal space derived from sensory, proprioceptive, and visual input [14]. Indeed, it has been suggested that the LRJT implicates the body schema as accuracy and reaction time (RT) are affected by both the position of the participant's anatomical part in space and the number of movements necessary to position the participant's body part to conform to that seen on the image [20–23].

Imaging studies demonstrate that the LRJT is a complex mental task associated with activation of subcortical and cortical structures including frontal areas involved in working memory and attention, pre-motor areas, basal ganglia, cerebellum, and sensory integrative areas in the parietal cortex [23–25]. The LRJT therefore appears to be related to processes involved with cognitive function, sensorimotor integration, movement planning, and execution [19, 25].

Studies with persons experiencing pain associated with musculoskeletal injuries and conditions demonstrate changes in peripheral [26–30] and cortical sensory [31–33], motor [34–51] as well as cognitive processes [52]. Any or all of these processes may be associated with LRJT performance. For example, LRJT accuracy of shoulder images has been associated with a functional shoulder motor task in healthy subjects [53]. Tactile acuity has been associated with LRJT accuracy for the back but not the knee in symptomatic participants [54]. In healthy subjects Botnmark et al. (2016) found no correlation between two-point discrimination of the shoulder and LRJT performance [53]. We have recently demonstrated that participants with pain associated with musculoskeletal injuries and conditions of the wrist and hand had altered LRJT and motor performance that was associated with a measure of affective distress suggesting that pain related factors may also be involved in this task [10].

The objective of the study was therefore to determine which of the cognitive, sensory, motor, and pain related factors were associated with LRJT performance in symptomatic participants with pain associated with musculoskeletal injuries and conditions of the wrist/hand. We hypothesized that motor imagery ability and cognitive aspects assessed with the Stroop test would be associated with LRJT performance of images of the hands and feet. We also hypothesized that sensory, motor performance, and pain related factors would be more specifically related to images congruent with the area of pain. A better understanding of the factors associated with LRJT performance provides valuable information into the variability of study results and the necessity to consider sensory, motor, cognitive, and even behavioural factors in research and practice involving the LRJT.

## 2. Methods

This was an observational cross-sectional study. The protocol and procedures conformed to the Declaration of Helsinki. The study was conducted at the Hand Clinic at the Centre Hospitalier de l'Université de Montréal, Notre Dame Hospital between June and December 2017. Ethical approval was granted from the institutional review board (CÉR-CHUM 16.372). Participants for the study were recruited when attending the hand clinic for consultation with plastic surgeons specialising in wrist and hand disorders. Participants were screened in the waiting area to explain the nature of the study, the requirements for their participation, and eligibility. Participants were required to be 18 years and older, experiencing pain associated with musculoskeletal injuries and conditions of the wrist/hand in their right dominant side that impacted their activities of daily living, were able to follow instructions and answer questionnaires in English or French, and suffer from no known neurological condition that impacted cognitive function and no musculoskeletal injuries and conditions of the lower extremities. Verbal and written informed consent was obtained prior to the commencement of the study. Demographic and descriptive information including gender, age, education, diagnosis, symptom duration, areas of pain, and taking of pain medications was documented. Handedness was verified utilizing the Edinburgh Handedness Inventory [55].

### 2.1. Dependent Variable

The LRJT involved determining if images of hands and feet were of the left or right side utilizing the Recognise™ (Neuro-Orthopedic Institute, Adelaide, South Australia) software [8, 56, 57]. The LRJT involved a block of 40 images of hands and of 40 images of feet presented on a plain (vanilla) background, with a maximum duration per image of 5 seconds, on an 8-inch computer tablet. Images for feet were included given the variability in the literature of altered LRJT performance in noninjured/nonpainful areas [11, 12] and as a control. Participants were instructed not to move their hands or feet to assist in determining laterality and to answer “as quickly and accurately as possible” by depressing the left or right button on the tablet screen that matched the laterality of the image presented. Participants were given the chance to practice on 10 images before proceeding with the actual tasks. The order of the block of images of hands and feet was randomized across participants. Results were displayed for accuracy (percentage of correct responses) and Reaction Times (RT) (seconds).

### 2.2. Independent Variables

#### 2.2.1. Sensory Measures

The West Haven Yale Multidimensional Pain Inventory (MPI) [58, 59] was utilized to assess subjective pain and measure the impact of their condition on patients' activities of daily living. The MPI consists of fifty-one questions answered on a 7-point Likert scale. Subscales involve grouping of questions scored between 0 and 6. In addition to pain severity, the measure assesses pain interference, life control, and affective distress as well as participation in leisure, social, household, and work activities. It is a well-researched and utilized instrument in research. Participants were also asked if their pain was constant or intermittent.

Pressure Pain Threshold (PPT) was determined by using a digital pressure algometry (Wagner Instruments, Greenwich, CT, USA, model# Wagner FPX25). PPT was measured bilaterally on the palmer aspect of the first carpometacarpal joint and the hypothenar eminence lateral to the pisiform. The average of three trials was recorded [60, 61]. The order of assessment for the site and hand was randomized across subjects.

Tactile acuity was assessed with the Two-Point Orientation Discrimination (TPOD) task utilizing a hand-held caliper (Fowler, Model # 54-101-150-2, Newton, MA, USA) [62, 63]. The participants were blindfolded and asked to indicate if they felt one or two points of contact. When two points were indicated, they were required to state if the points of contact were oriented vertically or horizontally [64]. The test was performed in both hands over the hypothenar and thenar eminences. To attempt to control for pressure of application the caliper was held at the end and only the weight of the caliper head was utilized to apply pressure. Assessment was performed in ascending and descending order with separations between 4 and 14 mm. Two vertical and horizontal trials were performed at each site for each distance of separation. The distance at which the participant consistently had 3/4 correct responses for the thenar and hypothenar eminences was recorded [64].

Proprioception was measured by evaluating Joint Position Sense (JPS). JPS was performed in the same manner as described by Kalisch et al. (2012) where subjects were blindfolded and instructed to compare sizes of two polystyrene balls of different diameters placed in their hands. Three different diameter polystyrene reference balls (7.0, 8.0, and 9.6 cm diameter) were placed in the participant's left hand by the examiner. A second polystyrene ball, of seven possible different diameters (6.6, 7.0, 7.3, 8.0, 9.0, 9.6, and 10 cm diameter), was placed in the right (affected) hand. Participants were instructed to squeeze the polystyrene balls and then relax the tension to control for thixotropy effects influencing JPS [65]. They were not permitted to manipulate or turn the balls. The participants were required to verbalize if the polystyrene ball in the right hand was smaller, larger, or the same size as the reference ball placed in the left hand within 5 seconds. Therefore, 3 reference balls were compared to 7 different polystyrene balls of different diameters for a total of 21 comparisons [66]. The number of errors was recorded.

#### 2.2.2. Motor Performance Measures

Motor performance was assessed by dynamometric evaluation of strength performed utilizing a hand-held Jamar dynamometer (Sammons Preston Rolyan, Bolingbrook, IL, USA) following recommended protocols [67]. Participants were asked to squeeze the handle as strong as possible and were provided with verbal encouragement. Three trials were performed on each side, alternating from side to side. The maximum value was recorded. The reliability and validity of the this task have previously been documented [67].

Fine and gross motor function was assessed with the Purdue Pegboard Test (PPG) (Lafayette Instruments, Lafayette IN, USA, Model #32020A), a standard manual dexterity test commonly utilized in research and in clinical settings that involves placing pins in slots with their right hand, left hand, and both hands in 30-second time epochs. A total score consists of the aggregate of these three measures. Finally, participants perform the building of small assemblies involving pins, washers, and collars in a one-minute epoch. The PPG has been assessed for reliability and validity [68, 69].

#### 2.2.3. Disability Measure

Disability of the Arm, Shoulder and Hand questionnaire (DASH) was utilized to assess both symptoms and functional status in patients with upper extremity musculoskeletal injuries and conditions. It is a self-rated assessment with documented construct validity and reliability [70, 71]

#### 2.2.4. Confounding Measures Associated with LRJT Performance

Studies of the LRJT allude to attention/concentration and motor imagery ability as possible confounding factors explaining experimental results in LRJT studies [11, 72, 73]. Therefore, cognitive function was evaluated utilizing a modified Stroop test [74] with the Encephalapp application installed on an 8-inch computer tablet [75]. The task involved the words red, green, blue, or a neutral stimulus (number signs - ###) randomly presented and written in red, green, or blue colours. Participants indicate as quickly and as accurately as possible the colour in which the word or neutral stimulus was presented by depressing the keys at the bottom of the screen (Red, Green, and Blue). The keys indicating the colours were also randomized and not fixed in a specific order. The participants were given practice runs until they successfully preformed the task with 10 images without making an error. The time taken to perform 2 successful trials of 10 images without making an error was recorded. Motor imagery ability was assessed by the Movement Imagery Questionnaire–Revised Second version (MIQ-RS) [76].

### 2.3. Sample Size and Statistical Analysis

Sample size was predetermined based upon an *α* = 0,05, power (1-*β*) = 0,8, 6 independent variables, and a moderate effect size of 0.25 (corresponding to coefficient of determination values of roughly 0.3-0.4). The minimal sample size required was 61.

Statistical analysis was performed utilizing GraphPad Prism 7 (GraphPad Software Inc, La Jolla, CA, USA) and SPSS 24 (IBM Corporation, Armonk, New York, USA) statistical software. Normality of data was assessed by visual inspection of the data and D'Agostino Pearson normality test.

Differences between LRJT performance measures between hands and between feet were performed utilizing paired T-tests. Pearson correlation coefficients were performed between LRJT performance (Accuracy and RT) and the independent variables. Adjustments for multiple comparisons were made when necessary using the False Discovery Rate Benjamini-Hochberg procedure with an *α* <0.05 [77, 78]. To investigate if changes in LRJT performance accuracy could be attributed to slower RT in participants in the PAIN group (accuracy-speed trade-off), Spearman rho correlations were performed between LRJT Accuracy and RT.

Multiple Linear Regression models were performed for each of the dependent variables (LRJT accuracy and LRJT RT for the hands and feet) with the sensory, motor, and cognitive measures. Variables that have previously been found to be related to LRJT performance in some studies such as age, pain severity, symptom duration, motor imagery ability, and concentration/selective attention (Stroop test) and inserting different permutations of the independent variables were entered into the multiple linear regression models, the choice influenced by correlation coefficients values and relevance. Choice of best model and which variables to maintain was based upon minimizing of the mean squared error, including independent variables where the coefficients had p values below p=0.10 and had the highest R and R^2^ adjusted values. Models were checked for multicollinearity and homoscedasticity.

As pain medication was a strong and significant predictor in the multiple linear regression model for LRJT performance accuracy, the participants were divided into two groups, those taking pain medication (PainMeds) and those who had not taken pain medication (NoPainMeds). Nonparametric tests were performed on demographic, pain, and disability measures between groups. Paired comparisons were performed for LRJT Accuracy and RT between these groups. As some LRJT performance data violated the assumptions of homogeneity and equality of variance, Mann–Whitney U nonparametric tests were performed.

## 3. Results

Sixty-one subjects participated in the experiment (31♂, 30♀). Participants experienced pain associated with musculoskeletal injuries and conditions of the wrist and hand including postoperative fractures/amputation, tendinitis, first carpometacarpal osteoarthritis, Dupruytren's, trigger finger, and wrist sprains. Descriptive information is found in Table 1. The sample consisted of persons experiencing pain between 1 and 228 months and therefore was comprised of persons with acute and chronic pain. The majority of participants had pain for greater than 3 months (56/61). Thirteen subjects took pain medication on the day of the evaluation. Twenty-nine participants described their pain as constant.

### 3.1. LRJT Right Hand Accuracy and Reaction Time

**Figure 1:**
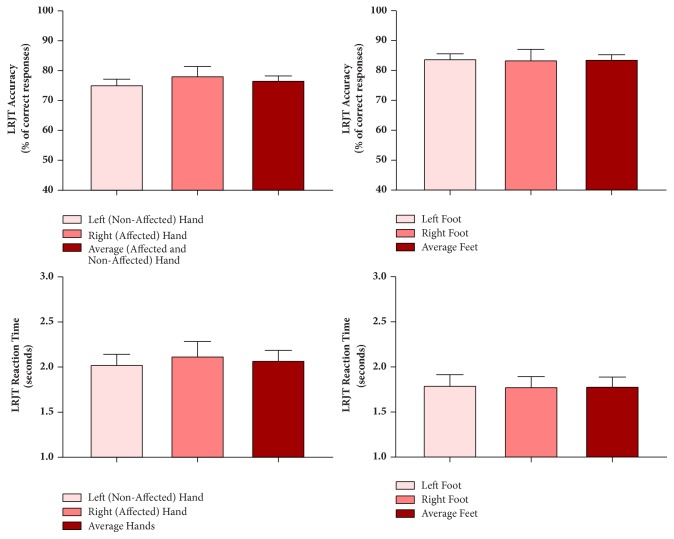
Left Right Judgement Task (LRJT) performance in participants with musculoskeletal disorders of the wrist/hand. Mean±95% Confidence Intervals.

No difference was found in LRJT accuracy or RT between hands and between feet (see Figure 1). Spearman correlation coefficients between LRJT Accuracy and RT were -0.29 for the hands and -0.37 for the feet. As the correlations were negative decreased reaction times were associated with increased accuracy suggesting that there was no accuracy-reaction time trade-off.

### 3.2. Multiple Linear Regression (MLR) Models

#### 3.2.1. MLR LRJT Right (Affected) Hand Accuracy

The best fitting MLR model (F_2,56_=4.11, p=0.002) included pain medication, MPI General Activities, Two-Point Orientation Discrimination of the Right Hypothenar, and Purdue Pegboard values of the left hand and, after entering Stroop test and motor imagery ability scores, accounted for an additional 20% of explained variance (R^2^ adjusted) (see Tables 2 and 3).

#### 3.2.2. MLR LRJT Left Hand Accuracy

The best fitting MLR model (F_4,54_=5.71, p=0.001) included pain medication, MPI General Activities, and Purdue Pegboard values of the left hand and after entering Stroop Test and Motor Imagery Ability scores accounted for only an additional 4% of explained variance (R^2^ adjusted) (see Tables 2 and 3).

#### 3.2.3. LRJT Right Hand Reaction Time

The best fitting MLR model for LRJT Right Hand RT (F_2,56_=4.42, p=0.017) included only the variables Stroop Time and Gender (see Tables 4 and 5).

#### 3.2.4. LRJT Left Hand Reaction Time

No statistically significant model could be produced with LRJT Left Hand RT entered as the dependent variable.

#### 3.2.5. LRJT Feet Accuracy and Reaction Time

Multiple linear regression models using LRJT Feet Accuracy and RT as the dependent variables explained (R^2^ adjusted) 27-35% of the variance and the Stroop time and MIQ-VMI scores accounted for 78% and 86% of the explained variance of these models.

### 3.3. PainMed versus NoPainMed

**Figure 2:**
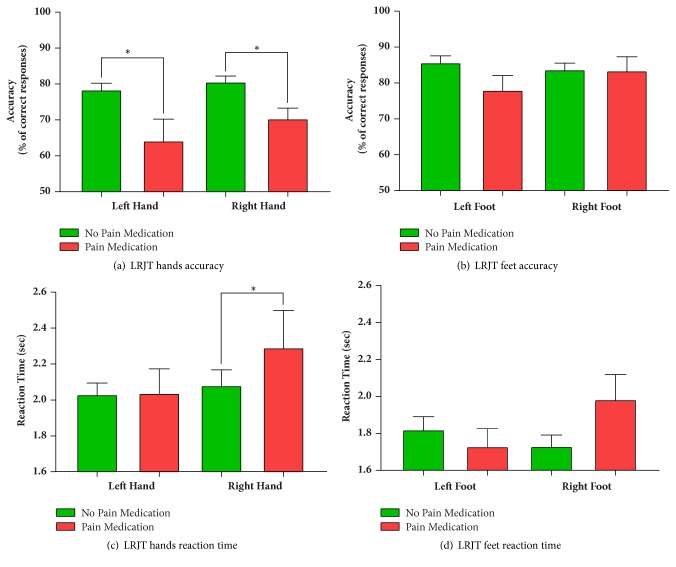
Left Right Judgement performance accuracy and reaction time in participants who were taking pain medication (PainMeds) and not taking pain medication (NoPainMeds) on the day of the evaluation. $\overset{-}{x}$±95% Confidence Intervals. *∗* False Discovery Rate Statistical significance below *α*=0.05.

LRJT performance was compared for the data of two groups, those who took pain medication (PainMeds) (n=13) (10 participants: acetaminophen, 2 participants: Lyrica, and 1 participant: Tramadol) on the day of the evaluation and those who did not (NoPainMeds) (n=48). A difference in LRJT accuracy between the two groups was found for the right hand (see Table 6 and Figure 2). LRJT performance values were lower in the participants who had taken pain medication on the day of the evaluation.

There was no difference in age, gender, or symptom duration between these two groups. After controlling for multiple comparisons, motor functions and Stroop test times were significantly different between groups. Purdue Pegboard Both Hands score was lower in the PainMed group ($\overset{-}{x}$=7.62±1.01) compared to the NoPainMed ($\overset{-}{x}$=11.09±0.40) group (p=0.002, U=114.5). Stroop times were greater in the PainMeds group ($\overset{-}{x}$=42.9±2.33) than the NoPainMeds group ($\overset{-}{x}$=35.8±1.08) and were statistically significant (p=0.013, U=151.0). Participants in the PainMeds group ($\overset{-}{x}$=3.82±0.32) had higher pain severity scores (p=0.020, U=177.5) than the NoPainMeds group ($\overset{-}{x}$=2.89±0.16).

Several variables including DASH scores and having constant pain demonstrated trends for differences between groups but were not significant after controlling for multiple comparison tests performed. Self-reported disability DASH scores were higher in the PainMeds group ($\overset{-}{x}$=54.25±6.40) than the NoPainMeds group ($\overset{-}{x}$=40.10±2.22) (p=0.04, U=171.0) and participants in the PainMed group ($\overset{-}{x}$=0.77±0.12) were more likely than the NoPainMed group ($\overset{-}{x}$=0.42±0.07) to indicate that they had constant pain (p=0.03, U=202.0). Affective distress values were greater in the PainMeds group ($\overset{-}{x}$=3.31±0.33) than the NoPainMeds group ($\overset{-}{x}$=2.64±0.19) but was also not statistically significant (p=0.08, U=209.5).

## 4. Discussion

The LRJT is utilized in rehabilitation as a method of treatment. However, there is variability in study results evaluating LRJT in participants with pain associated with musculoskeletal injuries and conditions and clinical measures associated with LRJT have not been investigated. We hypothesized that LRJT performance would be related to cognitive factors. We found that motor imagery ability and a measure of cognitive function, the Stroop test scores, explained a significant portion of the explained variance in the linear regression models of the LRJT. Secondly, we hypothesized that sensory, motor, and pain related factors would be specifically associated with the presentation of images of the right affected hand and would explain the majority of the variance in the model. Importantly, sensory and motor processes, the taking of pain medication, and participation in social, work, leisure, and household activities were responsible for 86% of the explained variance in the linear regression model for LRJT accuracy in the right, affected hand only. Novel and unexpected findings are that participants who indicated that they had taken pain medication on the day of the evaluation performed more poorly in the LRJT and that activities and participation were positively associated with better LRJT performance in the affected hand only.

### 4.1. LRJT, Age, Pain Severity, Symptom Duration, Motor Imagery Ability, and the Stroop Test

The LRJT is believed to involve implicit motor imagery where the participant makes an initial impression of laterality and then mentally imagines moving their hand in the same position as the image, and then either confirming or rejecting their initial impression of laterality [18]. Some studies involving the LRJT have found that factors such as age, pain severity, symptom duration, and gender are related to LRJT performance although results are variable. In the present study, age, pain severity, and symptom duration were only weakly correlated with LRJT performance. These were not included in the MLR models as motor imagery ability and Stroop tests resulted in better models based upon the criteria presented in the Methods section. A few studies have found LRJT performance to be related to pain severity [8, 18, 79] and with history of chronic back pain [79]. However, results of the present study found pain severity and symptom duration to be weakly associated with LRJT performance. This is in line with several other studies of LRJT performance in persons with pain including knee osteoarthritis [54], low back pain [16, 54], carpal tunnel syndrome [12], wrist/hand pain [10] as well as in persons with phantom limb pain and complex regional pain syndrome [15].

The belief that the LRJT involves implicit motor imagery is based upon at least two experimental findings. Imaging studies involving the LRJT demonstrate a similar pattern of activation as motor imagery [24, 80]. Secondly, the time to imagine the task is similar to the time to execute the task [81]. However, there appears to be some variability in the ability of persons to perform motor imagery [82]. When evaluating the use of motor imagery as a tool to enhance motor performance, motor imagery ability is associated with improved performance [76]. Therefore, it is unsurprising that motor imagery ability was correlated with LRJT performance and explained a significant portion of the variance in all the models except LRJT accuracy in the right hand.

The ability to perform the LRJT also requires complex mental processes. This is supported by imaging studies that demonstrate the activation of distributed cortical structures including those involved in working memory/attention such as the dorsolateral prefrontal cortex [24, 80]. The Stroop test is believed to be a measure attention and working memory but is also considered a measure of executive function and appears to involve cognitive flexibility, processing speed, and the ability to inhibit cognitive interference (see [83]). The inclusion of the Stroop test scores in the regression models for LRJT performance may therefore reflect changes in any of these measures of cognitive function. Not only does pain appear to affect cognitive processes [84], chronic pain is associated with structural and functional changes in the brain areas associated with these cognitive processes [52]. Which cognitive factors are specifically related to LRJT performance will require further study.

Although the majority of studies utilizing the LRJT have not controlled for motor imagery ability and cognitive factors such as concentration/attention, the present results suggest that such control is necessary when attempting to understand the different processes involved in LRJT performance including improvement in the task and differences between groups with and without pain.

### 4.2. LRJT, Sensory, and Motor Function

A measure of sensory function, TPOD, was also included in the linear regression model of LRJT performance accuracy in the right affected hand only. Stanton et al. (2013) previously found a correlation between two-point discrimination thresholds and LRJT accuracy in participants with back pain, but not in subjects with knee osteoarthritis [54]. Botnmark et al. (2016) found no association between LRJT performance and two-point discrimination of the shoulder in healthy participants. Two-point discrimination has been found to be correlated with organisation in S1 [85] and therefore may be associated with processes involved in sensorimotor integration. In light of the present results in symptomatic patients, tactile acuity appears to be one of several variables that are correlated with LRJT performance in the affected area.

In a previous study we found a stronger relationship between LRJT performance and Purdue Pegboard Test scores in the healthy control group [10]. The present findings, in a larger sample, found linear regression models with LRJT accuracy entered as the dependent variable in both hands had stronger correlations with motor performance of the left, unaffected, hand of the Purdue Pegboard Test. Purdue pegboard scores were higher for the left ($\overset{-}{x}$=13.2±3.48) than the right side ($\overset{-}{x}$=12.44±2.49) contrary to normative values that tend to be higher on the dominant side in healthy particpants [86]. Botnmark et al. (2016) found a significant negative correlation between LRJT RT and motor performance in healthy subjects. It is possible that the influence of motor function on LRJT performance is stronger on the uninjured side and healthy subjects.

### 4.3. LRJT, Pain Medication, and General Activities

Two interesting findings were the inclusion of Pain Medications and MPI General Activities subscale in the linear regression models for LRJT right (affected) hand accuracy. The regression model and subsequent nonparametric tests found that participants who reported taking pain medication on the day of assessment performed more poorly on the LRJT Hand accuracy. It is possible that taking the pain medication was simply a function of increased pain scores and that pain severity is associated with the poorer LRJT performance. However, the link between pain severity and LRJT performance is unclear with several studies finding no association [9, 12, 16, 54]. Furthermore, the weak/moderate correlation between pain severity and LRJT performance (R=-0.21 to 0.01) makes this unlikely. Alternatively, it can be argued that pain medication may influence cognitive function. The taking of acetaminophen has been associated with changes in cognitive function including error detection [87]. We are unable to discard the possibility that differences in LRJT performance between participants that did and did not take pain medication are attributed to the effect of analgesics on cognitive function. However, the finding that LRJT performance differences were largely specific to the affected hand is suggestive that the impact of taking of pain medication may not be attributed to a generalized effect of pain medication on cognitive function.

The participants who took pain medications demonstrated several differences with the participants who had not taken medication. Participants who took pain medication had greater pain severity, poorer motor function, and Stroop test scores and describing their pain as constant was close to statistical significance. Differences between nociceptive and neuropathic pain on central nervous system changes have previously been attributed to the differences between these two types of pain and the belief that neuropathic pain is more constant and unrelenting [44, 88]. Further identification of these factors may help to determine those persons who would benefit from the inclusion of cognitively driven rehabilitation strategies in addition to conservative rehabilitative treatments [5].

LRJT Hand accuracy performance was also positively correlated with the MPI subset of general activities. This subset is comprised of 18 questions related to the participation in household, work, leisure, and outdoor activities. There was no correlation between MPI General Activities and pain measures. Although speculative, increased activities and participation may help to maintain the integrity of the Body Schema of the injured area through use. Another possible explanation is that participants involved in greater activities and participation have higher self-efficacy. Self-efficacy is defined as the confidence in performing/managing a particular behaviour and in overcoming barriers [89]. In participants with fibromyalgia, greater self-efficacy for pain and function significantly predicted physical activity measured with the Arthritis Impact Measurement Scale, Physical Function (including mobility, physical and household activities as well as activities of daily living) explaining greater variance than demographics, disease severity, and psychological distress [90]. In women with hand osteoarthritis, multiple linear regression found self-efficacy as the most significant predictor of performance measured with the Canadian Occupational Performance Measure comprising subsections related to self-care, productivity, and leisure [91]. Further research is required to understand these relationships.

### 4.4. Limitations

All experiments were performed in a single setting. The study included participants who were experiencing pain associated with musculoskeletal injuries and conditions of the right dominant hand and may not be generalizable for the left hand. The participants who had taken pain medication was a small sample and a larger study would help to confirm these results. Although the TPOD is a more rigorous method of evaluation as it decreases the nonspatial cues that are associated with the two-point discrimination task, it has not been assessed for reliability and therefore results involving this measure of tactile acuity should be interpreted with caution. The adjusted R^2^ values for the multiple regression models did not explain the majority of the variance and therefore other variables are also implicated in the LRJT performance not included in the models.

## 5. Conclusion

The study has several important implications for rehabilitative research and practice involving the LRJT. The LRJT appears to be a multidimensional task that is related to sensorimotor but also cognitive processes. LRJT accuracy in the right affected hand of participants with pain was related to measures of cognitive, sensory, and motor function. These differences in sensory, motor, and cognitive function need to be addressed when attempting to understand differences in LRJT performance between groups. Differences in LRJT performance between a subset of participants suggest that taking pain medication, higher pain severity, impaired cognitive function, and decreased motor performance may be indicators of altered sensorimotor integration and highlight persons that may benefit from cognitively oriented rehabilitation strategies in addition to conventional rehabilitative care.

## Figures and Tables

**Table 1:** Participant descriptive information.

	Mean	Standard Deviation
Age (years)	55.82	13.57

Symptom Duration (months)	43.68	45.79

West Haven Yale Multidimensional Pain Inventory (max scores – 6)		

Pain Severity	3.09	1.17

Pain Interference	3.11	1.38

Life Control	3.88	1.24

Affective Distress	2.79	1.27

General Activities	2.69	0.95

Disability of Arm, Shoulder and Hand (DASH)	42.98	17.62

Pressure Pain Thresholds (kg)		

Right (affected) Hand	6.93	3.83

Left (unaffected) Hand	8.42	4.89

Two Point Discrimination (mm)		

Right (affected) Hand	10.92	2.89

Left (unaffected) Hand	10.16	2.72

Joint Position Sense (errors)	3.82	1.51

Purdue Pegboard Scores		

Right (affected) Hand	12.44	3.48

Left (unaffected) Hand	13.20	2.29

Both Hands	10.43	3.30

Total	35.31	9.50

Assemblies	21.89	7.86

Grip Strength (kg)		

Right (affected) Hand	23.60	13.35

Left (unaffected) Hand	30.65	14.20

Stroop Time (seconds)	37.25	7.99

Motor Imagery Questionnaire (maximum score - 98)	67.34	23.94

**Table 2:** Multiple linear regression models for Left Right Judgement Task Accuracy for the hands.

**LRJT ** **Accuracy**						**Change Statistics**				
		**R**	**R** ^**2**^	**Adjusted ** **R** ^**2**^	**Std. Error ** **of the ** **Estimate**	**R** ^**2**^ **Change**	**F ** **Change**	**df1**	**df2**	**Significant ** **F Change**
Right (affected) Hand										

	1	0.26	0.07	0.04	13.09	0.07	2.105	2	56	0.131

	2	0.57	0.32	0.24	11.61	0.25	4.819	4	52	0.002

Left (unaffected) Hand										

	3	0.46	0.21	0.18	15.67	0.21	7.353	2	56	0.001

	4	0.55	0.30	0.25	15.03	0.09	3.430	2	54	0.040

1. Predictors: (Constant), Motor Imager Questionnaire Visual Motor Imagery, Stroop Time.

2. Predictors: (Constant), Motor Imager Questionnaire Visual Motor Imagery, Stroop Time, Pain Medications, MPI General Activities, two-point orientation discrimination hypothenar right hand, Purdue Pegboard Test left hand.

3. Predictors: (Constant), Motor Imager Questionnaire Visual Motor Imagery, Stroop Time.

4. Predictors: (Constant), Motor Imager Questionnaire Visual Motor Imagery, Stroop Time, MPI general activities, Purdue Pegboard Test left hand.

**Table 3:** Coefficients of best fitting MLR LRJT Right Hand Accuracy.

LRJT Accuracy							Confidence Intervals (95%)	
		Unstandardized Coefficients B	Standard Deviation	Standardized Coefficients Beta	t	p	Lower Bound	Upper Bound
**Right (Affected) Hand**								

**1**	**(Constant)**	69.75	13.49		5.169	0.000	42.71	96.78

	**Stroop Time**	-0.06	0.24	-0.04	-0.252	0.802	-0.55	0.43

	**MIQ VMI**	0.29	0.17	0.25	1.686	0.097	-0.05	0.63

**2**	**(Constant)**	53.61	18.70		2.867	0.006	16.09	91.13

	**Stroop Time**	0.24	0.23	0.15	1.039	0.304	-0.27	0.71

	**MIQ VMI**	0.07	0.17	0.06	0.381	0.705	-0.28	0.40

	**Pain Medications**	-8.34	4.14	-0.26	-2.017	0.049	-16.64	-0.04

	**MPI General Activities**	3.36	1.85	0.24	1.818	0.075	-0.35	7.07

	**TPOD Hypothenar Right Hand**	-1.12	0.57	-0.24	-1.965	0.055	-2.26	0.02

	**Purdue Pegboard Left Hand**	1.39	0.73	0.24	1.815	0.075	-0.15	2.92

**Left (unaffected) Hand**								

**3**	**(Constant)**	74.70	16.15		4.625	0.000	42.35	107.06

	**Stroop Time**	-0.470	0.29	-0.22	-1.613	0.112	-1.05	0.11

	**MIQ VMI**	0.48	0.20	0.31	2.331	0.023	0.07	0.88

**4**	**(Constant)**	43.43	21.19		2.050	0.045	0.96	85.91

	**Stroop Time**	-0.34	0.29	-0.16	-1.183	0.242	-0.92	0.24

	**MIQ VMI**	0.24	0.22	0.15	1.084	0.283	-0.20	0.67

	**MPI General Activities**	4.73	2.33	0.26	2.030	0.047	0.06	9.40

	**Purdue Pegboard Left Hand**	1.74	0.94	0.23	1.844	0.071	-0.15	3.62

MIQ VMI: Motor Imagery Questionnaire–Visual Motor Imagery; MPI: West Haven Yale Multidimensional Pain Inventory; TPOD: Two-Point Orientation Discrimination.

**Table 4:** Best fitting multiple linear regression LRJT Right Hand Reaction Time.

					**Change Statistics**				
	**R**	**R** ^**2**^	**Adjusted ** **R** ^**2**^	**Std. Error of ** **the Estimate**	**R** ^**2**^ **Change**	**F ** **Change**	**df1**	**df2**	**Significant F ** **Change**
1	0.41	0.17	0.14	0.63	0.17	5.738	2	56	0.005

1. Predictors: (Constant), Gender, Stroop Time.

**Table 5:** Coefficients of best fitting MLR LRJT Right Hand Reaction Time.

						**Confidence ** **Intervals** ** (95**%**)**	
	**Unstandardized ** **Coefficients ** **B**	**Standard ** **Deviation**	**Standardized ** **Coefficients** **Beta**	**t**	**p**	**Lower ** **Bound**	**Upper ** **Bound**
(Constant)	0.74	0.43		1.726	0.090	-0.13	1.59

Gender	0.33	0.17	0.24	1.961	0.055	-0.01	0.66

Stroop Time	0.03	0.01	0.39	3.117	0.003	0.01	0.05

**Table 6:** Nonparametric test results for Left Right Judgement Task performance accuracy and reaction time in participants who were taking pain medication and not taking pain medication on the day of the evaluation.

	**Hand**				**Feet**			
**LRJT**	**Accuracy**		**Reaction Time**		**Accuracy**		**Reaction Time**	
	**Left**	**Right**	**Left**	**Right**	**Left**	**Right**	**Left**	**Right**
**P-value**	0.001*∗*	0.003*∗*	0.191	0.009*∗*	0.066	0.880	0.730	0.092

**Mann Whitney U**	125.5	143.5	386.0	460.0	208.5	303.5	292.5	407.5

**Test Statistic**	-3.31	-2.99	1.31	2.61	-1.84	-0.15	-0.35	1.69

*∗* False Discovery Rate Statistical significance below *α*=0.05.

## Data Availability

The data used to support the findings of this study are available from the corresponding author upon request.

## Abbreviations

DASH: Disability of the Arm, Shoulder and Hand
JPS: Joint Position Sense
LRJT: Left Right Judgement Task
MIQ: Motor Imagery Questionnaire
MIQ-VMI: Motor Imagery Questionnaire–Visual Motor Imagery
MLR: Multiple linear regression
NoPainMeds: Participants who did not take pain medications on day of evaluation
MPI: West Haven Yale Multidimensional Pain Inventory
PainMeds: Participants who took pain medications on day of evaluation
PPG: Purdue Pegboard Test
PPT: Pressure pain threshold
RT: Reaction time
TPOD: Two-Point Orientation Discrimination.
